# O.6.3-2 Implementation of a systems approach for co-creating actions to promote vocational students’ physical activity and wellbeing

**DOI:** 10.1093/eurpub/ckad133.282

**Published:** 2023-09-11

**Authors:** Clara Heinze, Anne Sidenius, Anne-Louise Bjerregaard, Rikke Fredenlund Krølner, Charlotte Demant Klinker

**Affiliations:** Health Promotion Research, Copenhagen University Hospital – Steno Diabetes Center Copenhagen, Herlev, Denmark; National Institute of Public Health, University of Southern Denmark, Copenhagen, Denmark; Health Promotion Research, Copenhagen University Hospital – Steno Diabetes Center Copenhagen, Herlev, Denmark; Steno Diabetes Center Zealand, Holbaek, Denmark; clara.heinze@regionh.dk; National Institute of Public Health, University of Southern Denmark, Copenhagen, Denmark; Health Promotion Research, Copenhagen University Hospital – Steno Diabetes Center Copenhagen, Herlev, Denmark

## Abstract

**Purpose:**

Participatory systems approaches are suggested to address the complexity of health promotion in communities but has not been tested and adapted to school settings.

The Data-driven Health Promotion at Vocational schools (Data Health) study aims to test and evaluate a systems approach to create collective actions to promote health among students. Three Group Model Building (GMB1-3) sessions were applied at eight vocational schools, with involvement from the local community, to develop a causal loop diagram (CLD), prioritise evidence-informed actions, and develop implementation strategies. This process evaluation aimed to determine if the GMB sessions were implemented as intended across intervention sites.

**Methods:**

According to the Medical Research Council guidelines, six components were assessed: recruitment, reach, fidelity, dose delivered and received and context.

Data included registration and observation notes from the workshops (n = 24) and recorded and transcribed qualitative interviews with the school programme-coordinators (n = 8). All data were analysed thematically.

**Results:**

At all intervention sites GMB sessions were completed in 2022, with representation of students, school staff and managers, the municipal staff and local community actors. Based on local data, four intervention sites chose to target physical activity and four sites chose wellbeing. At most intervention sites, attendance (reach) from all participant groups fell below expectation. Especially recruitment of school staff (average no. of participants n = 1.75; range 0-3) and managers (average no. of participants n = 0.63; range 0-2) was challenging, with lack of time as the main reason for non-attendance. The expected 'dose' of the GMB sessions was delivered to participants, whereas fidelity, conceptualised as consistency in delivery, was mixed due to contextual circumstances e.g. school size.

The school programme coordinators were generally positive about the GMB sessions but indicated mixed opinions about the value of CLDs to guide the discussion in this setting.

**Conclusions:**

The Data Health study is the first to apply a systems approach to health promotion among vocational students and adapt GMB methodologies to school settings. Understanding the implementation of a systems approach in a school setting provides vital knowledge for researchers and practitioners regarding enablers and barriers of this approach.

**Funding Sources:**

The Danish Regions, Tværspuljen, Helsefonden.

